# Tackling the Challenges of 21^st^-Century Open Science and Beyond: A Data Science Lab Approach

**DOI:** 10.1016/j.patter.2020.100103

**Published:** 2020-09-17

**Authors:** Michael J. Hollaway, Graham Dean, Gordon S. Blair, Mike Brown, Peter A. Henrys, John Watkins

**Affiliations:** 1 UK Centre for Ecology and Hydrology, Lancaster Environment Centre, Lancaster, UK; 2 School of Computing and Communications, Lancaster University, Lancaster, UK

**Keywords:** DataLabs, data science, virtual, collaborative, transparent, multi-disciplinary, big data

## Abstract

In recent years, there has been a drive toward more open, cross-disciplinary science taking center stage. This has presented a number of challenges, including providing research platforms for collaborating scientists to explore big data, develop methods, and disseminate their results to stakeholders and decision makers. We present our vision of a “data science lab” as a collaborative space where scientists (from different disciplines), stakeholders, and policy makers can create data-driven solutions to environmental science's grand challenges. We set out a clear and defined research roadmap to serve as a focal point for an international research community progressing toward a more data-driven and transparent approach to environmental data science, centered on data science labs. This includes ongoing case studies of good practice, with the infrastructural and methodological developments required to enable data science labs to support significant increase in our cross- and trans-disciplinary science capabilities.

## Highlights

• Offer a vision of data science labs as open, collaborative platforms in the cloud
• Discussion of how data science labs support open and transparent science
• Discussion of experiences around implementing data labs in practice
• The definition of a roadmap of research challenges around virtual data labs

## The Bigger Picture

As we move toward the need for open and cross-disciplinary science, and with an ever-increasing volume of data, there is a critical need to provide research platforms that support the wide variety of users that need to gain knowledge from this data. We present our concept of a “data science lab” as a key contribution in this area. A data science lab is a collaborative, dynamic, and tailorable platform that caters for users at varying levels of abstraction. We illustrate the concept with an initial implementation of a data science lab, drawing on our experiences in the cross-disciplinary field of environmental data science, thus aiming to develop a more data-driven and transparent approach to science. We set out a research roadmap to serve as a focal point for the international research community to take the concept forward and enable data science labs to support the ever-increasing requirement for cross- and trans-disciplinary science capabilities.

## Graphical Abstract

**Figure:**
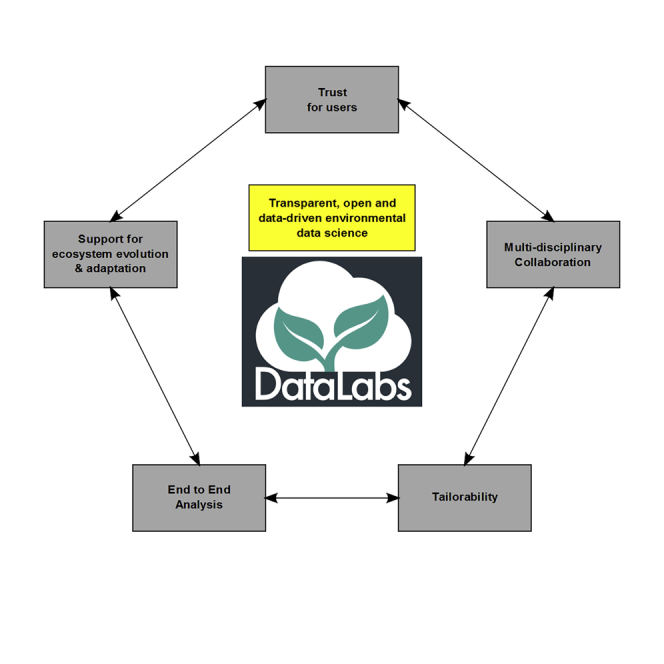

## Teaser

We present the “data science lab” concept as a potential solution to champion cross-disciplinary and open science. Data science labs are cloud-based, collaborative, and tailorable platforms enabling users with different requirements and expertise to find data-driven solutions to a wide range of environmental challenges. We present examples of methodological and infrastructural developments using data science labs along with a detailed research roadmap to serve as a focal point for developing a more data-driven and transparent approach to environmental data science.

## Introduction

With the widespread use of digital technologies in modern research and the rise of data-driven research, the nature of scientific discourse is changing to include the complete digital record of how scientific discoveries were derived. This has been mainly driven by demands to allow more open scrutiny of the scientific evidence underpinning policy decisions in response to perceived loss of public trust in scientific consensus.^1^^,^^2^ National science funding agencies are increasingly requiring openness and transparency in research they fund^3^ and scientific journals are increasingly requiring publication of digital materials alongside any manuscript.^4^ This move to “open science” has been championed by leading scientific agencies as the next stage of scientific discourse in a digital age, to increase the transparency and access to the scientific evidence on which important societal decisions are based (both in the public and private sectors). This is seen as an important principle for scientific endeavor to enable modern (often digital) social discourse as laid out in the open access policies of several national funding bodies (e.g., The Royal Society,^5^ the European Commission European, through the Open Science Cloud,^6^ the National Science Foundation,^7^ and the Chinese Academy of Sciences^8^).

Together with the pressure for open science has come the requirement for a more holistic approach to major research questions, such as responses to climate change, limits of ecosystem resilience, sustainability of agricultural practices, and impacts of policy trade-offs in management of natural resources and human health. This is especially true when providing scientific evidence to support policy development for sustainability goals, e.g., the United Nations Sustainable Development Goals^9^ at regional or national level. National science funding agencies, such as UK Research and Innovation^10^ have prioritized funding for larger, multi-disciplinary consortia in order to promote this more holistic approach in the provision of scientific evidence to government funders (as set out in the Nurse review of the UK research councils^11^). Such initiatives are also being adopted by other international agencies to support large multi-disciplinary projects (e.g., the EcoCloud initiative in Australia^12^).

Alongside these cultural developments has been the relentless increase in digital materials involved in scientific research commonly referred to as “big data” and characterized by a rapid increase in the volume, velocity, and variety of data being used. This has led to the rise of data science as an important discipline to facilitate scientific discovery from rapidly increasing “big data” resources.

We believe that these trends in the practice of scientific research, together with societal expectations of openness and scrutiny of scientific evidence, has led to the need for flexible, collaborative research environments, where researchers from different disciplines with highly varied skill sets can explore and learn from the wealth of data available using a range of data science and other modeling methods. This encourages publication of the full range of different data and methods that support an assertion, rather than a single analytical result that is vulnerable to being presented as irrefutable truth. To disseminate and evaluate these publications requires access to virtual, scalable computation resources that are seen as a trusted, collaborative workspace for research teams learning to work with each other as well as with new methods and data to produce a new quality of scientific outcome. Development of collaborative research environments are needed to underpin this change in working culture for data-intensive cross-disciplinary projects and facilitate a significant increase in science capability. This includes harnessing the power of data science methods and facilitating seamless access to such techniques to enable scientists and decision makers alike to extract meaning from ever-increasing datasets.

This paper introduces one such potential solution, a concept known as a data science lab approach. The overall goals of this work are as follows:

1) Define our vision of a virtual, cloud-based, collaborative, and transparent environment, and how they provide a vital platform for the future of open and transparent science.
2) Set out a clear and defined research roadmap on how we feel is the path forward in this rapidly emerging scientific field.
3) Provide a focal point for the research community to progress the cultural changes required for open and collaborative research.

The remainder of the paper is laid out as follows. We first present an overview of the current state of the art in infrastructure technologies that are trying to facilitate open research, such as virtual research environments (VREs). We then present our vision of the data science lab concept and how they will take open science forward. Finally we discuss the remaining gaps to be addressed and present a research roadmap to make data science labs a vital tool for championing open science.

## Results

### State of the Art

In this section we provide an overview of the current state of the art in the area, including related efforts to engineer solutions to foster transparent and collaborative scientific research. Historical development and the use of differing nomenclature for these environments are discussed, followed by a review of previous research visions and related research impacts. We then briefly cover some existing data science labs in different domains demonstrating their wide applicability across other scientific domains. Support and promotion of open science has been a key feature of recent developments and we describe a number of approaches and their supporting infrastructure. Finally, we describe some areas of interest in the engineering of these tools and in their adoption and sustainability.

Candela and colleagues^13^ note that the terms VREs, Science Gateways, Collaboratories, Digital Libraries, and Inhabited Information Spaces have all been used to describe environments where scientists can access data, software, and computational resources from a web browser. The vision described is that VREs will be integrated into standard working practices through phases of definition, deployment, and maintenance. Three major issues are identified in realizing the vision: large-scale integration and interoperability, sustainability, and adoption.^13^ Interoperability is important as they advocate an approach that explicitly suggest not trying to develop all resources from scratch and make use of existing approaches. Sustainability and adoption are inherently linked and contribute enormously to the success or otherwise of a VRE. The suggestion is that effort should be focused on community development processes, such as through awareness training and targeted engagement, rather than technology development processes.

Barker and colleagues^14^ illustrate how work on science gateways (interpreted broadly) has had considerable research impact with a growing number of conferences, initiatives, and journal special issues. The benefits reported include lowering barriers to computational infrastructure, enabling collaborations, sharing of resources, promotion of open science, and support for cross-disciplinary research. However, they also state various challenges remain in the areas of interoperability and data management, evaluation (specifically for incentives encouraging open science, reproducibility, and data and software citation) and in building the necessary skills and funding sources for longer-term sustainability.

Buddenbohm and colleagues^15^ develop and discuss a set of success criteria for VREs. They identify 12 criteria that can be applied from the perspective of a user, operator, or funder of a VRE and can be used as a template for developing success criteria for a specific instantiation of a VRE. The criteria include, for example, usage, knowledge transfer, collaboration, dissemination of expertise, reuse of infrastructure, scalability, and incorporation into existing workflows.

Data science labs have been used in many different application domains and many different communities. The Science Gateways Catalog^16^ lists nearly 600 entries and covers a broad range of subjects, including philosophy, mathematics, social science, and the physical sciences, with examples of applications promoting open science, including HUBzero^17^ and Agave.^18^ There are nearly 200 Science Gateways covering the Earth Sciences, including the Earth System Grid (providing access to data, models, and tools^19^) and NEON (a continental-scale ecological observation facility^20^). The BCCVL (The Biodiversity and Climate Change Virtual Laboratory)^21^ facility in Australia is a good example of a virtual laboratory. Its focus is on biodiversity and climate change and provides datasets, models, and experimental protocols. It also runs online courses on species distribution modeling and workshops that are integrated with university curricula. Within Europe, the EVER-EST project^22^ is looking to enhance research and capacity building in the Earth Sciences and has developed a VRE for research life cycle management for the Earth Science community.

One of the key drivers for data science labs is the provision of support for open science. Assante and colleagues^23^ identifies that technology support will be fundamental in delivering a vision of open science. This vision includes better interpretation, understanding, and reproducibility of research activities and results, enhanced transparency in the scientific life cycle, and a reduction in the overall cost of research. The approach they take is by integrating a social networking collaborative environment with a shared workspace, an open data analytics platform, and a catalog enabling effective discovery, access, and reuse of research artifacts solutions, thus allowing the realization of open science practices to be achieved.

Providing tools for the large-scale data-intensive open science we envisage requires support from the underlying infrastructure. The VRE4EIC program^24^ has proposed the e-VRE reference architecture for VREs that defines three logical tiers in research infrastructures (resource access, interoperability, and application services) and incorporates collaboration and communication facilities to improve research communication. The major contribution of this work is that it recognizes that in future we will want to make use of federated VREs and will need a systematic framework for this to happen.

The engineering of a data science lab as an open, interoperable system may be supported through appropriate architectural choices and the use of appropriate metadata for resources. Emami Khoonsari and colleagues^25^ describe an approach to delivering a data analysis system for metabolomics through the use of a microservice architecture deployed on-demand as a set of containers (Docker) using an orchestration framework (Kubernetes). The role of microservices alongside on-demand resource allocation for VREs is identified in Capuccini and colleagues,^26^ where a development methodology is described. The underlying principles include the use of continuous integration/continuous deployment (CI/CD) as a VRE collaboratively evolves, using infrastructure-as-code mechanisms for infrastructure provision and automated deployment tools for VREs.

The seamless integration of data science labs resources is addressed by Martin and colleagues^27^ through the use of unified catalog for resource metadata using X3ML mappings for schema mapping, data transformation, and aggregation. They highlight the future role of machine learning support for ontology matching, the use of linked data for describing resources, and the importance of workflows generating provenance information. Edwards and colleagues^28^ identified a number of lessons for using semantic information to describe resources. Insisting that users provide a lot of data for provenance purposes is likely to fail and a more relaxed, lightweight system will provide more useful information. Requests from people for metadata rather than systems works better and visualization of provenance metadata is useful.

Data science labs need to support sophisticated analytics across a wide range of data types. Mechanisms for data discovery, data integration, scalable analytics, and processing of novel data types, such as real-time streaming data are needed. Examples of approaches to these challenges are giving below.

Dimitrov and Stoyanov^29^ highlight that discovery of specific research data is not well catered for using traditional search engines and that some data will be confidential and not publically indexed. They describe a custom solution using metadata and based upon the open-source CKAN system using the SOLR search engine and a PostgreSQL database. It allows for searching via multiple terms, including keywords, partial phrases, research area, and communities. Data integration is also a considerable challenge and De Giacomo and colleagues^30^ survey approaches to this using ontology-based approaches. In general three components are used: (1) an ontology providing a high level representation of a domain, (2) existing data sources, and (3) a mapping between the two layers. They identify a number of challenges in using ontology-based data access methods. These include integration of non-relational data sources, evolution of ontologies over time and development of methodologies for ontology use alongside improved tool support.

An important aspect of data-intensive science is supporting scalable data analysis. This connects closely with the types of architectural support described above using cloud-based resources and in reducing the barriers to accessing these resources. Capuccini^31^ expands on previous work specifically covering microservices and integrates this with a large-scale machine learning framework using Spark and big data analytics using a MapReduce approach. New forms of data, such as streaming data now need to be integrated into scalable data analysis. For example, a sensor network provides an ever-increasing real-time dataset that needs to be collected, processed, and stored for monitoring and analysis. This presents a further engineering challenge to update and run services and analytics in real-time as the latest data streams in. Filgueira and colleagues^32^ coupled Apache Kafka/Spark, ElasticSearch, and the Python Falcon framework to create an Internet-of-Things processing hub based upon a microservices architecture. They plan to further extend the system adding RDF storage and querying and the use of Jupyter notebooks.

Effective use of VREs requires that users trust the services offered and that they address legal and regulatory requirements. Yin and colleagues^33^ identify two classes of trust-related requirements. The first covers privacy, security, trust, and legal requirements and specifically identifies the need for precision and clarity in legislative compliance and the approaches taken to meet these requirements. The second focuses on data provenance, highlighting “pathways of data” and data publication information. More generally, systems have to earn trust from users in their relation to data and data access, and through the usability and stability of the software provided.

### The Data Science Labs Concept

In this section, we draw upon our experiences from existing multi-disciplinary “environmental data science” projects to present our concept of the collaborative data science lab framework. The major issues a data science lab must tackle are to champion open science into the future, deal with extracting information from the explosion in big data, and provide a collaborative platform that supports multi-disciplinary research groups that are ever present in environmental data science. Firstly, we present our vision on the key features a data science lab must consider in order to address these issues. We then introduce our concept of a data science lab and present examples of how they deliver the required features, including presenting an exemplar case study in detail.

A new cross-disciplinary, data focused, integrated, and transparent way of working presents a number of technical and cultural barriers that current approaches do not address and must be overcome. We propose that the engineering of collaboration and openness in data science labs is crucial to foster the new mode of scientific practice required to break down these barriers. Therefore, we set out the following vision for key attributes that need to be considered in potential solutions (Table 1).

**Table 1:** Key Features Desired in Moving toward Open, Transparent, and Big Data Science, Including Key Areas that Need Addressing

Key Feature	Key Focus
Collaboration	An environment must be provided that brings experts, stakeholders, and decision makers from different domains into a single space where they can develop, access, and execute analytical routines and visualize the results. This includes supporting users of different technical levels to enable dissemination of scientific outcomes
Tailorable	The resources must be flexible and tailorable to a varying range of challenges and research questions. A user should be able to populate a lab with different data, methods, and computational resources as they require
End-to-end analysis	The workflows must provide end-to-end support for an analytical process from data ingress through to visualization/presentation of final results
Support for ecosystem evolution and adaptation	The environment must be able to integrate rapidly with the underlying infrastructure to enable ready development of new features based on evolving user requirements. Furthermore, the environment must be able to adapt to constantly changing resource requirements and optimize processing (e.g., integration with distributed computing resources for processing heavy tasks)
Brokering trust	The environment must act as a trusted broker in facilitating access to the underlying data and methods. There must also be efficient recording of provenance in the system to ensure data and workflows meet the FAIR standards

Our implementation of a data science lab (herein termed DataLabs) is a consistent and coherent cloud-based environment that champions open and collaborative science and decision making by providing the infrastructure and platform to bring scientists (from different disciplines), stakeholders, policy makers, and the public into one space to tackle a range of scientific problems. They provide an environment that supports end-to-end analysis from the assimilation and analysis of data through to the visualization, interpretation, and discussion of the results. Existing VREs and Scientific Gateways provide this ability to a certain extent; however, they are often domain specific or require a high level of expertise of the underlying infrastructure to engage with the problem at hand, thus providing a barrier to some users. We see DataLabs as a tool that draws on existing technologies (e.g., notebook technology and cloud computing environments) to realize our concept of a collaborative and multi-disciplinary environment that caters for many different levels of user abstraction within environmental data science research teams. DataLabs have a focus on tailorable and cyclical analytical workflows that evolve based on user requirements and iterative discussions between domain and methods expertise. They also harness the power of the cloud environment in which they sit to provide a common research environment with seamless access to high level compute and storage. Therefore, we see DataLabs delivering the desired attributes set out in Table 1 as follows.

#### Collaboration

**Figure 1:**
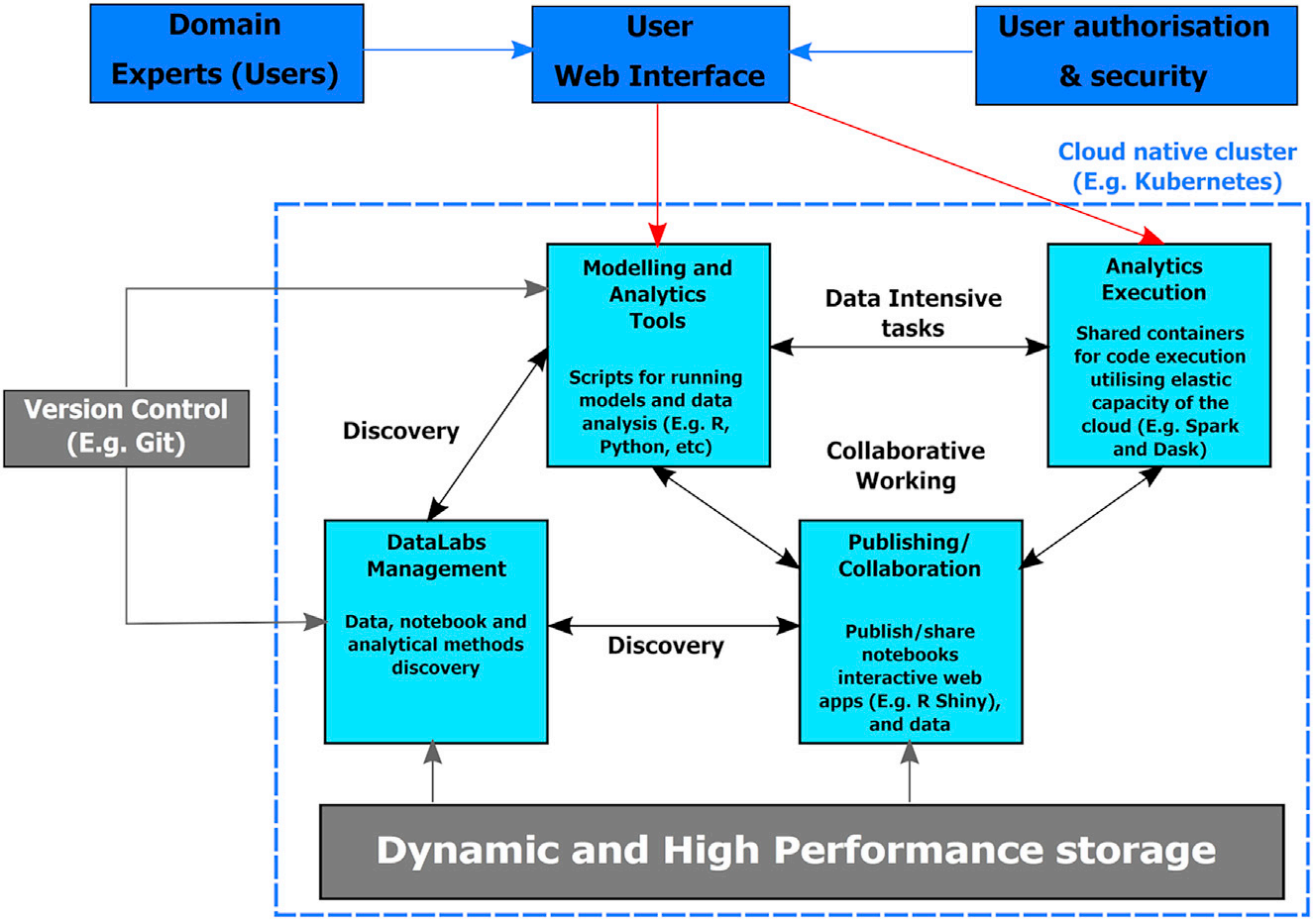
Schematic Overview of a DataLab Highlighting Key Infrastructure and Features to Promote Collaborative and Open Science in a Trans-disciplinary Cloud-Based Workspace

DataLabs champion collaboration and enable provenance through version control and change documentation of analytical workflows either through high level interfaces or through exposing underlying code, depending on the end user's experience (Figure 1). A key feature of a DataLab is the different levels of complexity at which a user can engage with analyses. This can range from developing and editing code using a notebook environment (with support for a number of commonly used languages, such as R, Python, and Fortran) through to visualizing the output either as a series of data plots or using a graphical user interface (e.g., R Shiny). These different levels of abstraction allow communities with varying levels of coding experience to work in one space to tackle key challenges. Furthermore, this enables users to have easy access to new data science methods for analysis, which is critical in the age of big data. Finally, package management systems for R and Python (e.g., Conda and Packrat) enable documentation of the environment used (e.g., package versions) to produce a coherent environment for user to collaborate and execute workflows in.

#### Tailorable

DataLabs can be developed to focus on a varying degree of research questions leading to the creation of different types. These can range from those that focus on a particular model or dataset or those that focus on a particular location (e.g., the Eden river catchment or London air quality). Labs can also be focused around the application of a specific method, such as changepoint analysis or extreme value theory. The containerized focus of the infrastructure enables the setup of adaptable methods and workflows that are flexible and tailorable depending upon the user's data, and analytical and computational requirements. This can range from incorporating new data into the lab to bringing in different methods through swapping-specific code cells within a notebook or bringing in another notebook altogether. Containerization of the Labs (or indeed parts of it) enables a particular code base or method to be ported to another cloud provider should the user require extra resource (e.g., cloud bursting) or allow the analysis to be executed elsewhere should the user wish. This includes persistent package management (including versions of particular libraries) using both conda and packrat utilities to maintain a coherent analysis environment across systems. This further demonstrates the generalizability of workflows developed in the labs and the ability to adapt to the constantly evolving requirements of environmental data science.

#### End-To-End Analysis

The notebook environment (Figure 1) of DataLabs provides the potential to harness a wide range of environmental data, analytical methods, and assessment and visualization tools. More importantly, the use of notebook technologies provides the potential to record the end-to-end provenance of a particular workflow. This enables end users to understand the reasoning behind decisions made in the analytical processes and allows them to reproduce or adapt the methods utilized. Notebook technologies can also document the processes used to ingest or access the data (either through an application programming interface [API] or directly) and any assumptions made during processing of the data. Finally the methods utilized to enable export of the results from the lab can also be recorded. This enables the documentation of the end-to-end workflow as a more cyclical approach whereby a domain-specific scientist interacts with the workflow at various stages and influences its development using an iterative approach. This approach is enhanced through the support for ecosystem evolution in the DataLabs architecture (see below) and further enhances the tailorable nature of the workflow to various challenges.

#### Support for Ecosystem Evolution and Adaptation

The DataLabs architecture is built as a set of composable services using a cloud native design philosophy. Individual services are developed as containerized applications, which are then deployed, managed, and scaled through a container orchestration platform. This approach supports the rapid development of new features, via loosely coupled self-contained services, which can be integrated into the infrastructure using CI/CD practices and support of the orchestration platform. This supports our vision that a DataLab will be dynamic in nature and evolve over time based on the challenge at hand. A lab may start out focusing on a particular method but may gradually become oriented around a particular project/location as new analytical methods and processes are incorporated. Therefore, as data and computational processing needs change the storage and computational services can evolve as required using cloud-based services to ensure optimal use of available resources.

#### Brokering Trust

DataLabs are able to serve the role as a trusted broker in facilitating access to data and the underlying methods by ensuring that the workflows developed support the FAIR (findable, accessible, interoperable, and reusable) data standards. Integration with package management systems (see above) as well as versioning of environments through git repositories allows users to reproduce the environment used to execute a particular workflow further ensuring FAIR standards are met. The project structure within DataLabs allows users to specify who can access the project folder, including access to the underlying data stores and notebooks for analytical methods. This is maintained through credential-based access to labs themselves (through authentication and authorization services), along with project level user privileges maintained by the project owner(s). This enables data privacy of sensitive nature stored within the DataLabs themselves. Furthermore, the use of APIs can be provided to allow access to limited or aggregated forms of sensitive data without exposing the raw data itself, either within or outside of the platform depending on where the data are stored. In addition, the labs will be integrated with version control (currently being implemented) in order to provide provenance and transparency in the analytical process allowing, decisions, parameter values, and code versions used to be recorded at each stage of the workflow. This enables other users to reproduce or reuse aspects of the workflow as necessary in their own projects. Finally, data privacy is ensured with user-based credential access to DataLabs themselves (Figure 1).

### Experience with DataLabs

In this section we provide an overview of our current experience in implementing DataLabs and how they have been utilized to deliver a platform that provides a number of the desirable attributes that we define in Table 1. Presently, this is mainly focused at using experience from the fields of data and computer science to address challenges with an environmental science focus. A wide variety of use cases exist (Table 2) ranging from those which look into big data analytics (robust indicators of habitat extent and condition) to large data ingress and storage (EMEP4UK) and flexibility of methods to variable datasets (state change of long-term data). All of the use cases demonstrate the collaborative nature of the DataLabs framework and the feasibility of them being adapted to a wide range of different challenges within the environmental data science domain. We now focus in detail on one use case as an exemplar of where DataLabs can champion open science into the future. This particular example focuses on a novel approach to model evaluation using changepoints analysis (last example in Table 2).

**Table 2:** Summary of Current Use Cases for DataLabs

Topic	DataLabs Description	DataLabs Technical Challenge	DataLabs Key Feature Addressed
Robust indicators of habitat extent and condition	Integration of high quality, but sparse, ground survey data with high coverage, but with potential classification errors, through remote sensing of derived data. This analysis is used to provide unified national estimate of the extent of four key habitats	Big data analytics with the integration of the Spark distributed processing framework on a computer cluster to reduce lengthy analysis times	• Support for ecosystem evolution and adaptation • Collaboration
Species distribution	Analysis of trends in unstructured occurrence datasets	Development of SPARTA (species presence absence R trends analysis) packages and use on the Spark distributed processing framework	• Support for ecosystem evolution and adaptation • Collaboration
Environmental DNA	Use of environmental DNA (eDNA) sequence data to calculate relative abundance of diatom species (and other algae) and relate to water quality measures	Development of a microservice architecture to allow service composition for different software pipelines. This provides a multi-experimental platform to compare across different choices of algorithms, reference databases, and water quality indices	• Collaboration • Tailorable • End-to-end analysis
State change of long-term data	Provide more contextual information for quality assurance of long-term monitoring data stored at data centers	Providing the flexibility to implement state change algorithms across a large variety of datasets regardless of format, sub-disciplines, and owners. Because it is for QC, it does not need to be very accurate but fast enough for users to quickly decide whether to use a dataset or not	• Tailorable • Collaboration
EMEP4UK	Atmospheric chemistry transport model for UK hourly atmospheric composition at scales ranging from 100 to 1 km	Large (9TB) data ingress, storage, and access control using object storage. Distributed processing using Dask and data transformation to cloud native format (Zarr)	• Support for ecosystem evolution and adaptation • Collaboration
Crop-Net	Integrated crop modeling for different scenarios. Collaborate with different stakeholder engagement and integration of a wide range of data types and decision-making approaches	Integration of agile development processes for iterative design of a Data Lab and its user interfaces	• Collaboration • Support for ecosystem evolution and adaptation • End-to-end analysis • Brokering trust
Changepoint analysis	A new analytical method that combines changepoint analysis with fuzzy logic to evaluate the timing of changepoints between two different time series. This is applied in the context of evaluating performance of high-resolution climate models (15 km) against weather station data over the Greenland Ice Sheet	Integration of process-based and statistical models in a flexible framework that can be adapted to address different environmental challenges	• Collaboration • Tailorable • End-to-end analysis

These case studies each focus on a different environmental science focused challenge and show a range of different technological challenges that are faced. The key desired attributes that lab provides (see Table 1 for details) is also shown.

### Case Study: The Challenge

In environmental sciences, numerical models are utilized to forecast how the natural environment will respond to changes in key drivers and pressures (e.g., climate change). With the increasing availability of computational power, these models are becoming more complex and are operating at much higher spatial scales. One such area of focus is the application of regional climate models (RCMs) over the Greenland Ice Sheet in order to estimate the impacts of rising temperatures on processes that exhibit very high spatial variability (e.g., ice sheet melt and surface mass balance). These RCMs are typically used as interpolators to downscale general circulation model (GCM) output at relatively coarse resolution (∼79 km) to much finer scale (∼15 km) and therefore in theory better represent local scale processes.^34^ The process of evaluating such models involves the combination of large volumes of observational and model data along with the calculation of global metrics, such as mean bias or the Nash-Sutcliffe index. Furthermore, to gain better reasoning of performance for fine-scale events in both space and time, complex statistical methods (often written in specific coding languages, such as R or python) are usually deployed requiring input from experts from different domains. Finally, there is the requirement of communicating the results for interpretation by users of different levels of expertise. This often requires interactive methods to visualize and explore complex output. Often, this sort of exercise is done by passing data and analysis scripts from one expert to another working in different computational environments. This often results in many different points of entry, non-coherent environments, and no end-to-end record of assumptions made during the analysis. Therefore, new approaches are required to best facilitate such an RCM evaluation workflow.

### Case Study: The DataLabs Solution

In response to this challenge, a DataLab has been developed to allow the exploration and comparison of how well a GCM and an RCM capture “changepoints” in air temperatures on the Greenland Ice Sheet. In summary, a changepoint is a point in a time series where the properties of a statistical representation of that series (e.g., mean, variance, or trend) undergo significant change.^35^ This would indicate the potential occurrence of a key event that we would expect the RCMs to capture better than the lower-resolution GCMs.

**Figure 2:**
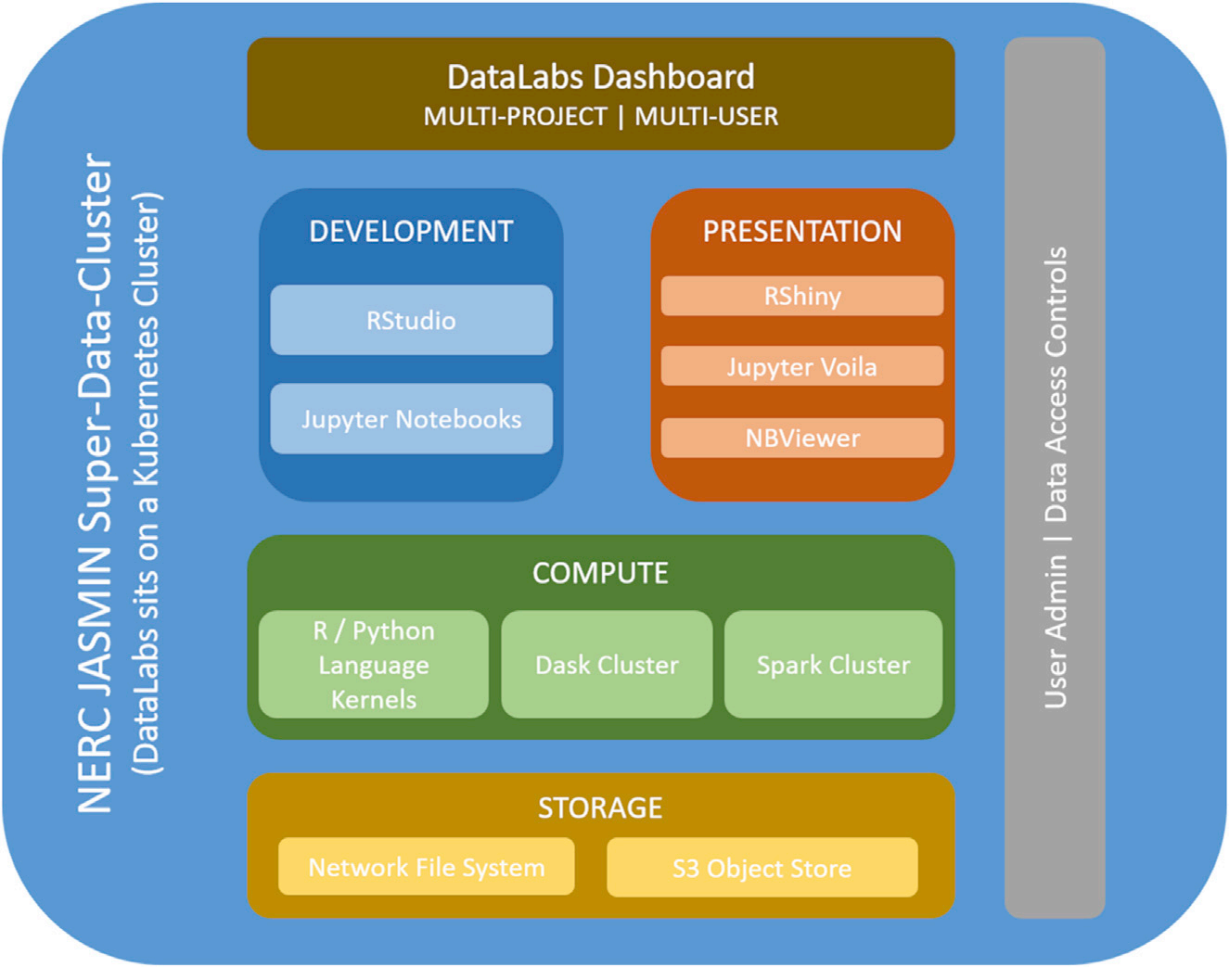
Schematic Overview of the Software Stack Being Deployed in the Changepoint DataLab Case Study

A schematic representation of the DataLab software stack is shown in Figure 2. The cloud-based environment (accessed through a Web-based dashboard interface) provides a storage volume that sits below compute, development, and presentation resources. These are all managed using cloud native technologies (such as Kubernetes) to provide a collaborative space in which to deploy and develop the changepoint analysis. The storage volume brings together observed temperature time series data from a suite of automatic weather stations (AWSs) with large-scale model output from both the GCM and RCM models (in gridded format). Access to this data store is available to all users of the DataLab ensuring each user is consistently working with a common data resource. The changepoint evaluation workflow itself is developed and set up in a notebook environment that is executed using the R programming kernel. The notebook reads in air temperature data from the AWSs from around the Greenland Ice Sheet along with the corresponding time series from a GCM model and an RCM model and bring these together with a suite of complex statistical methods to process the model evaluation.

**Figure 3:**
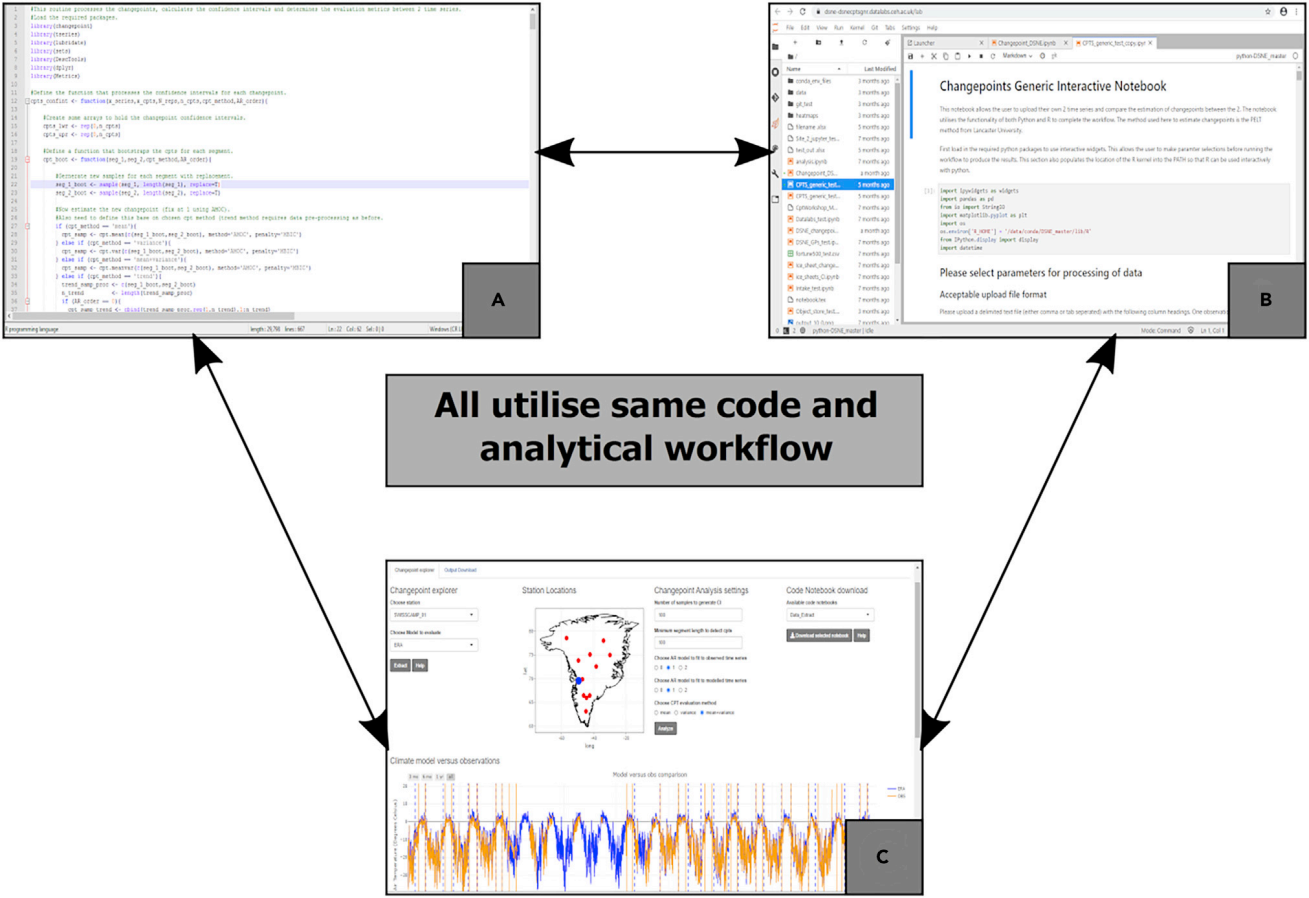
Overview of the Changepoint Case Study DataLab Demonstrating the Different Levels of Abstraction (A) The raw R code for computing changepoint locations. (B) The Jupyter notebook demonstrating the method. (C) R Shiny app to allow exploration of changepoints at different sites across Greenland.

The different levels of abstraction of the DataLab are demonstrated in Figure 3, whereby statisticians and environmental scientists can come together and develop or apply novel (or existing) methods using raw code (Figure 3A) or the collaborative notebook environment (Figure 3B). To raise the level of abstraction, an R Shiny app (Figure 3C) sits above the code to allow users of different levels of expertise to explore the changepoint analysis at all of the available sites across the Greenland Ice Sheet. Crucially, all of these different techniques for executing the analysis are operating over the same underlying code base, which ensures coherency across the different levels of abstraction, including parameter settings and assumptions made. The R Shiny app also allows quick exploration of the stations to identify common events across the network that the model(s) fail to capture. This therefore allows rapid visualization and dissemination of results to stakeholders and end users for interpretation without the need to view the underlying code. Functionality has also been provided for users to upload their own time series into the notebook (and R Shiny app) to allow exploration and comparison of event timings for a wide range of different time series. This demonstrates the transferability of the method using a common code base.

### Case Study: Delivery of Desired Key Attributes

The DataLab developed for this case study mainly focuses on delivering the following main attributes that are required (Table 1):

#### Collaboration

The lab environment provides a suitable platform for collaboration with users of different analytical experience. Firstly, the R Shiny app embedded in the notebook allows users with no code experience to critique the various climate models (or load in their own data if available) and evaluate the ability to capture changepoints. This platform also allows data scientists and environmental scientists to share their results with policy makers and other end users, while being transparent as to what assumptions were made in the analysis and why. This provenance is enabled through version control of the notebook using an interface with git. Further to this, the notebook environment of the lab fosters collaboration and enables new analytical workflows to be brought into the process to further critique the numerical models. For example, a user could incorporate extreme value analysis into the model evaluation framework. As the DataLab sits above a common data store, all new methods brought in maintain consistency by working with the same datasets in a coherent computational environment (e.g., using the same versions of available R and python libraries).

#### Tailorable

The notebook format of the changepoint example lab provides a detailed workflow of how the data are prepared for the analysis and how the changepoint methods are applied to each time series. Therefore, the method is easily transferable to other areas of environmental science (e.g., how well do models capture shifts in air pollutants concentrations). Further to this, should a new user wish to experiment with the comparison of different changepoint estimation methods, they can edit the changepoint code cell as desired. The lab presently only deals with 18 years' worth of daily data; however, should the user wish to apply the method to larger volumes of data, more models, or data at finer spatial scales, the computational elasticity of the cloud is available to handle the requirements. This is facilitated through the availability of distributed computing resources, such as Dask and Spark clusters (Figure 2). This scale-up can be demonstrated through a simple Dask Kubernetes example whereby a small cluster of eight nodes can process analysis on every cell of a grid that takes up 128GB of memory, compared with a 2GB array (in memory) in a non-parallel example.

#### End-to-End

The transparency of the lab environment also provides the reasoning behind the decisions made at each stage of the analysis to be made. This documents the entire process captured by the workflow from ingestion of the raw data to assumptions made during data manipulation through to parameter settings for any analytical methods and ultimate dissemination of the results. This allows scientists, collaborators, and end users alike to understand all assumptions made during the workflow which enables reproducibility of the analysis to be made should the user wish to utilize the method for another problem. For the case study presented here the following steps are demonstrated.

Step 1: data ingress into the lab as netcdf (model data) and csv (observed data) through file uploader.
Step 2: data wrangling: large netcdf file read in, geolocation of stations, extraction of data at stations, and processing into format to be used by analytical routines.
Step 3: execution of the changepoint analysis and processing of results.
Step 4: visualization of results in R Shiny application.
Step 5: egress of the results through a direct download from the Shiny app.

The case study builds upon simple execution of the analytical method, by also explicitly considering the ingress of data, processing of raw data, and dissemination of the results to domain specialists, along with the assumptions made at each stage. In this case, the end-to-end nature of the workflow is presented as a demonstration of the reproducibility of the analysis and takes on an iterative and cyclical approach based on input from domain specialists at different stages. For example, at the data ingress and wrangling stage (steps 1 and 2) the data engineers investigate solutions for getting the data into an appropriate format for analysis. The data scientists and statisticians work on the method development and execution stage (step 3) and share the results with the numerical modeler at the visualization stage. Finally the results are disseminated to decision makers and end users across different levels of abstraction through direct egress of the data or through the R Shiny application (step 5). Based on the inputs at various stages, the workflow is adapted based on the challenge at hand. This draws on the expertise and experiences of the computer scientists and is based around an agile CI/CD approach. This process can be facilitated by the R Shiny interfaces or within the code itself using notebook technologies or a combination of both. Finally, if methods, workflows, data engineering solutions, and examples of best practice are identified from a given DataLab, they can be incorporated into the infrastructure if required. This further demonstrates the tailorable and reproducible nature of the workflow and the co-development approach encouraged within the DataLabs environment.

#### Brokering Trust and Support for Ecosystem Evolution and Adaptation

The recording of the provenance of the workflow along with the integration with git ensures that the methods deployed in the lab meet the FAIR standards. This ensures the lab acts as a trusted broker for access to the underlying data, methods, and assumptions around the workflow. Finally, as the computational and data needs of the lab change or it evolves to be focused around a particular project rather than a method, the cloud native software stack can evolve easily to provide the additional resources required in the most optimal way.

As demonstrated above, the case study is set up drawing on the multi-disciplinary domain of environmental data science whereby data science methods are being used to tackle environmental science's grand challenges.^36^ The case study requires input from scientists from different domains and over a wide range of abstractions (from raw code through to Shiny applications). This includes statisticians and data scientists to develop the changepoint method, environmental scientists to interpret the results and assess the suitability of the method to the current challenge, and computational scientists to develop and provide the platform on which to serve the application(s). This highlights how the DataLabs platform can serve as a key tool in a future of open, transparent, and multi-disciplinary science.

## Discussion

### Gap Analysis

Previous work in the development of VREs has demonstrated many different research activities exploring different aspects of the concept. However, these have often focused on specific elements or individual challenges for the domain of interest only. What seems to be missing is work bringing together these activities into a coherent vision and identifying key components where further research work is required. The vision and desired key attributes we set out previously (Table 1) allow us to assess the current state of the art and highlight areas where there is work to do in achieving this overarching vision and meeting the challenges.

Firstly, it is necessary to break down the cultural norms of working in silos and encourage people to work more transparently in a collaborative space. This will require strong supporting mechanisms to establish and maintain trust when sharing data, programming code, and analytical frameworks. Support for different collaboration mechanisms will be required to support varying forms of sharing and interaction. In doing this, we also want to work alongside community working practices and not to enforce different ways of working. The transparency and collaboration we seek will come from supporting current practices and in providing higher levels of abstractions for using the underlying technologies in a way that promotes trans-disciplinary working.

Existing VREs tend to be tailored toward specific domains or problems (e.g., Nectar) or involve following complex workflows (e.g., Taverna) that require a high degree of computational knowledge to set up and use. Indeed NanoHub^37^ and the associated HUBzero^17^ platform are exemplars of existing environments in which a wide range of stakeholders are supported. These range from undergraduate projects to large research consortia. However, some degree of expert knowledge and compute resource is still required to utilize these platforms. Therefore, there is a need to provide more appropriate abstractions for different classes of users in the field of environmental data science (e.g., environmental modelers, domain experts, or policy advisors) and for different types of DataLabs (e.g., data exploration, method development, or decision making).

This tailoring for different classes of users and for different types of DataLabs should ideally be built upon a consistent and coherent environment that is configured on a per-project basis. This allows changes in underlying datasets, derived data products, and analytical methods to become available to other project users as required. Clearly this would require some degree of control and management of the visibility of underlying data and methods (possibly through integration with version management systems and continuous deployment approaches).

Moreover, there is the need for different types of underlying computational infrastructure to meet the demands of different data-intensive science. We would envisage project-specific application components (e.g., different types of programming language support, analytical methods, storage, and computational frameworks) to sit upon common underlying research infrastructure to support resilient and scalable research environments.

Supporting this, the growing interest in microservice architectures, containerized components, and cloud native applications and frameworks points the way for future developments of DataLabs as a set of services that can be configured and composed in different ways. This service-oriented perspective will support the transfer of knowledge developed in one domain to be encapsulated and made available in other domains. For example, analytical methods developed in the context of one domain, such as changepoint and extreme value analysis of ice sheets should be easily transferable to other application domains, such as air quality. This could draw upon experiences from existing VREs, such as VRE4EIC,^24^^,^^27^^,^^38^ which has individual components wrapped up as microservices (e.g., a workflow service or a metadata service). However, in the case of DataLabs a particular lab (e.g., changepoint analysis) could be served as a microservice or even sub-components of a lab, such as the data ingress workflow.

Another area of consideration is based around the development costs and maintaining the sustainability of DataLabs. The development of the current implementation of DataLabs has been supported through funding from the UK Natural Environment Research Council to deliver collaborative projects as part of key environmental data science projects. To date, this has utilized approximately 4,000 h of developer time, around 400 h of administration and user engagement at a cost of £550k over two phases. In addition, approximately 1.5FTE is required to maintain the DataLabs infrastructure, support existing features and implement new ones. Further to this, additional support has been utilized from UKRI-supported grants, such as the Data Science of the Natural Environment project for development of some of the use cases presented in Table 2. The resources required to deliver these projects are summarized in Table 3. The use of DataLabs has resulted in indirect costs savings by providing a simple user interface to the storage and compute provided by cloud-based infrastructure. This enables more efficient sharing of data and analytical workflows between scientists, stakeholders, and decision makers. Headline examples include EMEP4UK, which enabled users to easily query, access, process, and visualize 9TB of data when previously it was shared manually using USB media (Table 3). Another example is the Robust Indicators of Habitat Extent and Condition which involved utilizing the big data analytical capabilities of the DataLabs to reduce the execution time of the method from hours on a local machine to minutes within the DataLabs. However, in order to take DataLabs forward, the costs of further infrastructure development needs to be considered. Indeed, future collaborative projects wanting to use the platform are already costing infrastructure and development costs into grant bids in order to implement DataLabs of varying sizes depending on requirements.

**Table 3:** Summary of Resources Required to Set up the DataLabs Use Cases

Topic	Developer Effort (Days)	User Time (Days)	User Experiences
Robust indicators of habitat extent and condition	20	5	Easy access to compute and storage power of the cloud enabled reduction of lengthily analysis times from hours on local machine to minutes within the DataLabs environment
Species distribution	5	0.5	Setup of Spark cluster for previous example enabled rapid exploration of this method saving user compute time
Environmental DNA	90	20	Users went from a depending on a previously static analysis produced by an external organization to a flexible analytical platform where they could try different approaches. Cloud native platform enhanced analysis speed and enabled collaboration with stakeholders
State Change of long-term data	20	4	Enabled rapid prototyping to demonstrate different analytical methods for state change to serve as potential QC method for different datasets
EMEP4UK	30	5	Cloud native storage and compute enabled data pipelines to be developed to cut big data into manageable chunks for easier and more efficient access. Users are now able to easily query and visualize a 9TB file in DataLabs which was impossible previously
Crop-Net	30	3	Enabled rapid prototyping to demonstrate the effects of climate change on suitability for crop growth and their yields. R Shiny apps enabled easy sharing of results with stakeholders
Changepoint analysis	25	5	Enabled numerical modelers to easily access a flexible framework to combine statistical and process-based models with simple access to cloud-based compute for data-intensive analysis

The use cases are presented in Table 2. Each summary is presented in number of days required by developers and users to set up the use case.

It is clear in this discussion that there is a huge degree of interest both in open science, and the benefits to society that this could create, and in exploring approaches to this in providing collaborative research infrastructure and DataLabs. The question this poses is how best to make progress on realizing this vision?

### DataLabs: A Research Roadmap

Based on the previous sections, it is clear that there has been progress toward our vision of DataLabs, but a wide number of research questions remain unanswered. In this section, we present a research roadmap of key challenges that if addressed would greatly enhance the state of the art in this increasingly important area. The research roadmap is clustered into five complementary themes consistent with the desirable features of DataLabs set out in Table 1.

### Theme 1: Collaboration

To embrace a new style of environmental data science that is more open, collaborative, and integrative we must encourage community acceptance and uptake of DataLabs as a platform. We need to explore methods to build and maintain vibrant communities around DataLabs through supporting usability and the right level of abstraction. This is already being explored within DataLabs (using interfaces, such as R Shiny) as well as in other VREs, such as HUBzero. Furthermore, the correct underlying methods need to be explored to facilitate collaboration and shared access to environmental assets. Collaboration is already brokered using notebook technology and version control within DataLabs but further exploration is required on options to “publish” a particular DataLab to share with the wider community (e.g., through Binderhub) or the linking of DataLabs with a wiki to aide communication between users and developers.

As DataLabs are aimed at serving a variety of user groups that will include both method development and scientific discovery and support for decision making in environmental change, it is critical that results presented are meaningful for all stakeholders involved. As demonstrated through the case study, DataLabs presently harness the powers of notebook technologies and visualization dashboards (e.g., R Shiny) to foster collaboration between various stakeholders. However, further methods need to be explored to best utilize notebooks to support open, collaborative, integrative, and reproducible science and allow full demonstration of scenarios or complex analytical workflows. Furthermore, notebooks presently offer good capabilities for collaboration on the analytical front; however, other communication media need to be explored to bring in decision and policy makers. This leaves a vital question as to what visualization services should be provided in DataLabs (above and beyond dashboards)? Can we also embrace experiences from the arts, information sciences, and journalism disciplines to explore additional means of interpreting and presenting “data,” which is an intrinsically creative process? There is also the need to support decision making under uncertainty where there is a requirement to make said uncertainty visible and most importantly interpretable to both scientists and stakeholders.

### Theme 2: Tailorable

As environmental data are ever increasing in volume, veracity, and variety, it has become ever important to recognize the complexity involved in addressing analysis in both time and/or space. Furthermore, there is the ever-increasing need to move away from structured data and operate more flexible data representations that capture both structured and unstructured data. Currently DataLabs use a project structure for discovery of data and analytical methods; however, there is a need to explore appropriate data architecture options to allow users to discover and interact with the data. Data Catalogs and Semantic Web concepts (including linked data and ontologies) are potential options to support the complex and varied nature of environmental data.

The desired tailorability of DataLabs also needs to explore the advantage of its deployment on cloud infrastructure taking advantage of significant benefits offered in terms of the underlying elasticity and scalability in terms of computational resources when required. DataLabs already explore this within its current cloud environment using Dask/Spark to scale up analysis and the containerized (through Docker) nature of a DataLab allows it to be platform independent. This currently allows the potential to port a DataLab to another cloud provider if required, following similar approaches to other VREs (e.g., HUBzero). In addition, we need to explore options to engineer cloud brokers that can optimize the use of resources across multiple providers, including public, private, or indeed hybrid cloud systems.

### Theme 3: End-to-End Analysis

We recognize the importance of modeling in environmental data science and the significant benefits of moving models into the heart of DataLabs and the potential for them to co-exist with data science methods (e.g., statistical models, such as changepoints) or other styles of modeling (e.g., agent-based models). Such migration of models to the cloud and model coupling approaches require the documentation of the end-to-end analysis of the workflow. This is presently documented as a cyclical process in DataLabs (see Case Study sections) utilizing an agile approach. However, further options for documenting the provenance of the workflow need to be explored. This is particularly important to document assumptions made at each stage of the model coupling process. These principles also extend to any collaborative analysis undertaken in data labs from ingress of data to communication of results to support decision making.

### Theme 4: Support for Ecosystem Evolution and Adaptation

As DataLabs are a dynamic and tailorable resource we recognize that there is the requirement for the underlying software architecture to also be dynamic to support families of different DataLabs. These will have different capabilities, requirements, and durations of existence. Current tools and techniques within DataLabs include Docker, notebooks, and conda environments within Kubernetes to maintain and evolve existing DataLabs. However, the question remains whether instantiation of members of a given DataLabs family can be automated and interact with data and method discovery mechanisms. Dynamic DataLabs and the ability to integrate streaming data requires distributed systems that are often complex to manage and need to be scalable when processing heavy tasks are executed. Currently in DataLabs, users utilize Dask/Spark in a somewhat manual process to scale up analysis; however, there is the need to explore potential methods to automate this process as and when required by the analysis. Therefore there is a requirement to further explore adaptive or self-adaptive management of the distributed infrastructure to support the desired tailorability of the DataLab itself (Table 1).

### Theme 5: Brokering Trust

One of the key aspects of supporting a future of open and collaborative science is exploring how DataLabs themselves can enhance trust in the underlying methods, data, and science. Presently, DataLabs utilize the capabilities of notebook technologies integrated with version control to record the provenance of a particular workflow (including the cyclical end-to-end development process) in order to ensure that data and methods meet the FAIR standards. However, other methods could warrant exploration to further enhance this trust, including the potential to utilize blockchain technology in this context. Finally, the need to balance openness with ensuring a given level of security and/or privacy in specific instances of DataLabs needs to be explored. The current project structure, which allows sharing of particular notebooks and/or datasets between authorized users within a given DataLab goes some way to solving this. However, further options need exploring to share data and methods between instances while maintaining data privacy. APIs offer potential solutions whereby certain derived products from the data are exposed while protecting the raw data itself.

### Overall Reflections

It should be clear from the above research questions that the scope of this work is vast and hence there is a real need for collaboration to cover this space. Furthermore, the research agenda requires input from the environmental sciences, computer science, data science, social sciences, and creative arts (among others). Hence, it is crucial to build a fundamentally trans-disciplinary research community to realize our vision of DataLabs. Finally, to be effective in this domain, it is important to have a level of international consensus on approaches to supporting DataLabs, including agreeing on standards where appropriate, and this implies a strong level of coordination across the scientific community.

### Conclusions

This paper presented an overview of the challenges faced in moving to a future where transparent, collaborative, and multi-disciplinary science is taking more of a center stage. In this paper we present our vision of DataLabs, which we see as a key tool in bringing data, environmental, and computer scientists into a common and coherent environment. In such a space, they are able to work together to utilize their different expertise in order to champion data science solutions to some of environmental science's grand challenges. In addition to highlighting the significant progress already made in the development and application of DataLabs, this paper also sets out a clear and defined research roadmap on how we feel is the way forward in this rapidly emerging scientific domain. We believe this can form the focal point for an international research community progressing the cultural changes for open and collaborative science, ongoing case studies of good practice, and infrastructural and methodological developments required to enable DataLabs to support a significant increase in our trans-disciplinary science capabilities.

## Experimental Procedures

### Resource Availability

#### Lead Contact

Further information and requests for resources and reagents should be directed to and will be fulfilled by the Lead Contact, Michael Hollaway (mhollaway@ceh.ac.uk).

#### Materials Availability

This study did not generate new unique reagents.

#### Data and Code Availability

The code and documentation for the general implementation of DataLabs is available through GitHub (https://github.com/NERC-CEH/datalab).
